# Eye and Ear Temperature Using Infrared Thermography Are Related to Rectal Temperature in Dogs at Rest or With Exercise

**DOI:** 10.3389/fvets.2016.00111

**Published:** 2016-12-19

**Authors:** Brian M. Zanghi

**Affiliations:** ^1^Nestlé Purina Research, St Louis, MO, USA

**Keywords:** core body temperature, exercise hyperthermia, canine, brain temperature, infrared thermography

## Abstract

Rectal body temperature (BT) has been documented in exercising dogs to monitor thermoregulation, heat stress risk, and performance during physical activity. Eye (BT_eye_) and ear (BT_ear_) temperature measured with infrared thermography (IRT) were compared to rectal (BT_rec_) temperature as the reference method and assess alternative sites to track hyperthermia, possibly to establish BT_eye_ IRT as a passive and non-contact method. BT measures were recorded at 09:00, 11:30, 12:30, and 02:30 from Labrador Retrievers (*N* = 16) and Beagles (*N* = 16) while sedentary and with 30-min play-exercise (pre- and 0, 15, 30-min post-exercise). Total exercise locomotor activity counts were recorded to compare relative intensity of play-exercise between breeds. BT_rec_, BT_eye_, and BT_ear_ were measured within 5 min of the target time. Each BT method was analyzed by analysis of variance for main effects of breed and time. Method differences were compared using Bland–Altman plots and linear regression. Sedentary BT differed by breed for BT_rec_ (*p* < 0.0001), BT_ear_ (*p* < 0.0001), and BT_eye_ (*p* = 0.06) with Labs having on average 0.3–0.8°C higher BT compared to Beagles. Readings also declined over time for BT_eye_ (*p* < 0.0001) and BT_ear_ (*p* < 0.0001), but not for BT_rec_ (*p* = 0.63) for both breeds. Total exercise (30-min) activity counts did not differ (*p* = 0.53) between breeds. Time and breed interaction was significant in response to exercise for both BT_rec_ and BT_ear_ (*p* = 0.035 and *p* = 0.005, respectively), with a marginal interaction (*p* = 0.09) for BT_eye_. All the three methods detected hyperthermia with Labs having a higher increase compared to Beagles. Both BT_ear_ and BT_eye_ were significantly (*p* < 0.0001) related to BT_rec_ in all dogs with sedentary or exercise activity. The relationship between BT_eye_ and BT_rec_ improved when monitoring exercise hyperthermia (*r* = 0.674) versus measures at rest (*r* = 0.381), whereas BT_ear_ was significantly related to BT_rec_ regardless of activity (*r* = 0.615–0.735). Although BT readings were significantly related, method bias (*p* < 0.02) was observed for BT_eye_ to slightly underestimate BT_rec_, whereas no bias was observed between BT_ear_ and BT_rec_. This study demonstrates that IRT technology effectively measures both ear and eye temperature and enables effective monitoring of BT changes at rest, with exercise, and between breeds. However, ear, and not eye, temperature is a better reflection of rectal temperature.

## Introduction

Hyperthermia has been documented in dogs, in response to exercise at various durations and intensities, and is an important physiological measure of thermoregulation, heat stress risk, and a factor limiting performance during physical activity (1–4). These early characterizations of hyperthermia during and after exercise were largely examined through recording rectal temperature, but newer technologies like ingestible sensors (5–7) and non-contact infrared thermography (IRT) (8) are enabling sensitive and alternative methods of measuring body temperatures (BTs) to monitor hyperthermia. Although rectal BT measures are sensitive in detecting changes in thermoregulatory responses because of dehydration (1, 2) and body cooling (3, 9), the need to stop the dog to obtain a measure, or have a continuously placed thermocouple for recording during laboratory-based treadmill exercise, are minor disadvantages. More recent work in exercising dogs has demonstrated the advantages of using an indigestible telemetric sensor that is swallowed and records core BT during exercise on various sampling intervals as it passes through the gastrointestinal tract (5–7).

Alternatively, IRT is useful and routinely used by veterinary clinicians to measure auricular (ear) temperature (10, 11) but requires contact of the animal for probe placement, and some animals exhibit apprehensive behaviors during attempted measurement. IRT has also been employed as a passive and non-contact method of measuring surface temperature of a subject’s infrared radiation (12). Non-contact IRT is a promising technology that recently has been reviewed for its use in veterinary applications (13). Among other applications, non-contact IRT has been examined to successfully detect inflammatory conditions or fever in cows, horses, or ponies (14–16), as well as stress-induced hyperthermia in dogs (8). The research with ponies and dogs has demonstrated that IRT of the eye is a valid index to measure core BT and is sensitive to detect physiological fluctuations when compared to rectal temperature (8, 16).

Although IRT of the eye and ear temperature have been used to assess BT in a variety of canine research studies and clinical settings, the use of these two anatomical locations to assess the natural increase in BT with exercise has not been reported. The study was designed to examine BT of eye and ear in dogs of two different breeds (Labrador Retrievers and Beagles) at rest or following play-exercise using IRT technology compared to rectal temperature as the traditional standard method of measuring BT. Two different breeds of dogs were examined because BT differs with breed size (17). Ultimately, if the IRT eye method is successful, then the potential exists to use remote and non-contact IRT to monitor hyperthermia in the field in working dogs (possibly in real time) without disturbing work or performance.

## Materials and Methods

### Animal Care and Feeding

Thirty-two dogs (16 males and 16 females) of two different breeds; 16 Labrador Retrievers (Labs) and 16 Beagles were evaluated. The Beagles had an average age of 4.0 ± 2.4 years, average weight of 11.7 ± 1.8 kg SD, and body condition score of 5–6 on a scale of 1–9 (18). The Labrador retrievers had an average age of 5.2 ± 1.5 years, average weight of 29.3 ± 2.5 kg SD, and body condition score of 5–7 on a scale of 1–9 (18). All dogs used in the exercise trial were required to have overall good general health prior to beginning the trial and evaluated by a veterinarian on the morning of each exercise day.

All dogs were fed once daily to maintain body weight with *ad libitum* access to water. Dogs were maintained on existing food (Purina Pro Plan Sport Active 26/16 Formula Dry Dog Food, Nestlé Purina PetCare Company, St. Louis, MO, USA) through the duration of the trial. Dogs were given *ad libitum* access to water before the exercise challenge and immediately after the 30-min exercise. Dogs were housed indoors with natural lighting and exposure to natural light cycles in pens (1.5 m × 4.5 m; 2 dogs per pen). Dogs were grouped on the basis of age, compatibility, and sex. All dogs were housed at the same kennel location and could see other dogs in adjacent pens. All dogs had direct interaction and socialization with caretakers on a daily basis and had continuous access to multiple toys.

### Experimental Design and Play-Exercise

Body temperature of sedentary activity was measured on a day when the dogs were resting prior to any outside play time with indoor ambient temperature between 68 and 70°F, whereas measurement of BT on play-exercise days occurred 3 days later with a warm indoor ambient temperature between 78 and 80°F with 78% humidity. Sedentary activity consisted of normal indoor-only kennel activity during the 6-h BT recording period. Outdoor kennel access was provided after the last BT measurement was recorded.

To conduct the bout of play-exercise for all dogs on a single day, dogs were allotted into groups of four based on compatibility, and balanced for gender and breed. Play and subsequent data collection for a given play-group occurred within approximately a 1.25-h block of time, with group 1 beginning at 08:00. Group 2 began after the completion of group 1, etc. Exercise consisted of group play and interaction among the four dogs, which included voluntary running and playing, as well as interaction with familiar pet-care staff to encourage voluntary ball and/or toy chasing and retrieving if individual dogs were not playing with other playmates. Exercise occurred in a designated indoor play area (10 ft wide by 70 ft long) with multiple familiar balls and toys of various sizes and textures to facilitate play and retrieving.

### Body Temperature Measurements

Body temperature was determined using three measurement locations. Rectal body temperature (BT_rec_) was measured by insertion of a digital thermometer (Accuflex Pro, Model 016-639, Physiologic, Montreal, QC, Canada) approximately 1 cm to acquire an automated reading upon pressing the measurement button. Ear temperature (BT_ear_) of the tympanic membrane and ear canal was detected by infrared detection of both ears (Pet-Temp PT-300, Advanced Monitors Corp., San Diego, CA, USA). Dogs with hanging pinna had the pinna temporarily pulled back to allow access to the ear canal for immediate insertion of the device probe. Reading was obtained upon pressing the measurement button and recorded from the digital output screen. Eye temperature (BT_eye_) using a portable thermal camera (IR966 professional radiometric video camera, Sierra Pacific Innovations Corp., Las Vegas, NV, USA). BT_eye_ was determined from thermographic infrared images of each dog’s face (8) encompassing both the right and left eye to assess fluctuations in BT. Two images were captured once the autofocus feature established a clear image at a distance of approximately 1 m and same angle. All the images were analyzed using thermal imaging analysis software (SPI IrAnalyser version 5.0.21.95) where both the maximum and average temperature of each eye were calculated within a rectangular area traced around the eye to include the entire eyeball and approximately 1 cm around the outside of the eyelids.

For BT recorded when the dogs were at rest, all three BT measurement sites were recorded within 5 min at 09:00, 11:30, 12:30, and 02:30. For assessment of BT before and after exercise, all BT measurement sites were recorded within 5 min immediately pre-exercise, immediately (0-min), 15-, and 30-min post-exercise play-bout. Pre-exercise and 0-min post-exercise measures were determined while the dog was outside of its kennel and outside of the exercise area, whereas 15 and 30-min post-exercise measures were obtained when the dog was in its home kennel run.

### Locomotor Activity Recording

Locomotor activity counts were recorded for 24 h using the MotionWatch 8 activity monitoring system (CamNtech Ltd., Cambridgeshire, UK). The MotionWatch 8 is tri-axial accelerometer placed inside a specially designed case and attached to a collar around the dog’s neck the evening before the play-exercise day. Placement of the device on the collar permitted normal rest, exercise, and feeding activity levels to be measured (19). The devices recorded activity counts on a 30-s epoch setting. Following removal of the device from the dog’s neck, data were downloaded to a PC-based MotionWare Software (version 1.0.3, CamNtech Ltd., Cambridgeshire, UK), and data were exported as a MotionWare-generated “.mtn” file. Each “.mtn” file was converted to a text file with Notepad (version 6.1, Microsoft Corporation, Redmond, WA, USA), and activity data transferred to Microsoft^®^ Excel^®^ 2013 (Microsoft Corporation, Redmond, WA, USA) for alignment with epoch recorded time. Total locomotor activity counts were calculated for the 30-min duration of play-exercise.

### Statistical Analysis

All statistical analyses were conducted using PROC MIXED in SAS (SAS 9.3., Copyright© 2002–2010 by SAS Institute Inc., Cary, NC, USA). To characterize BT measurements obtained from the eye, ear, or rectum, a separate analysis of variance (ANOVA) was performed for each BT location to assess the main effects of breed and time, and interaction between main effects. *Post hoc* analyses were performed using a Protected (*p* < 0.05) Fisher’s LSD to separate means of dependent variable that differed with time or breed. Regression coefficients and prediction equations were generated by use of linear regression analysis. Paired *t* tests were used to assess differences in mean values for BTs, as measured *via* the various methods (eye or ear) versus the reference method (rectum) for Bland–Altman plot analysis. Significance was determined at a value of α = 0.05.

## Results

### Daytime Body Temperature Measurements

**Figure 1:**
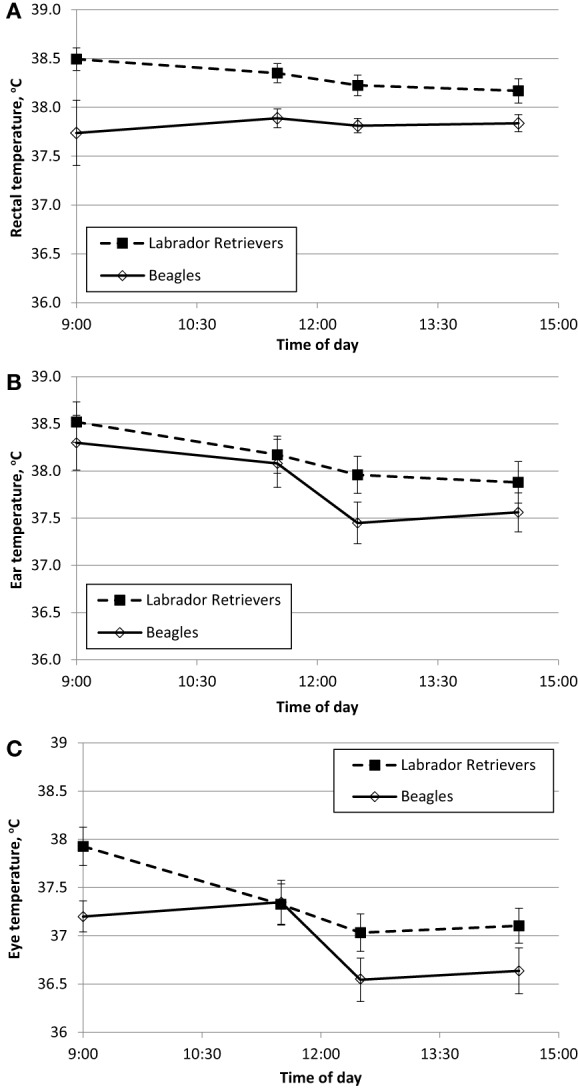
**Phase 1 mean (±SE) body temperature over time within a day in sedentary Labrador Retrievers or Beagles**. **(A)** rectal body temperature, **(B)** ear temperature, and **(C)** eye temperature.

To characterize resting daytime BT across multiple methods, measures were recorded at approximately the same time from the ear, eye, and rectum. BT_rec_ measures are used to establish “normal” daytime fluctuation at thermo-neutral indoor temperature between 20 and 21°C (68–70°F). BT in Celsius degrees is plotted over time for each breed with data obtained from rectum (Figure 1A), ear (Figure 1B), or eye (Figure 1C).

Temperature measures for each method were analyzed separately for ANOVA to evaluate the main effects of breed and time. BT_rec_ was measured as the reference value, and there was a significant effect of breed (ANOVA *p* = 0.001) with Labs having a mean temperature of 0.5°C higher (38.3 ± 0.1°C SE) compared to Beagles (37.8 ± 0.1°C SE). Labs were always numerically higher across all four daytime measurement occasions, this difference ranged from 0.3 to 0.8°C (Figure 1A). Neither the main effect of time (ANOVA *p* = 0.63) nor the interaction between breed and time (ANOVA *p* = 0.40) were significantly different.

BT_ear_ measures resulted in a significant effect of breed (ANOVA *p* = 0.02), as well as a main effect of time (ANOVA *p* < 0.0001). The interaction between breed x and time (ANOVA *p* = 0.39) was not significant. Mean BTear at 14:30 was approximately 0.7°C lower compared to 11:30, and Labs (38.2 ± 0.1°C SE) on average had a 0.3°C higher ear temperature compared to Beagles (37.9 ± 0.1°C SE).

For BT_eye_, there was a significant effect of time (ANOVA *p* < 0.0001), with afternoon measurement times being lower by approximately 0.8°C compared to morning times. There was also a marginal effect of breed (ANOVA *p* = 0.06) with Labs having a mean temperature of 0.4°C higher (37.3 ± 0.1 C SE) compared to Beagles (36.9 ± 0.1°C SE). The interaction between breed and time (ANOVA *p* = 0.24) was not significant.

### Comparison of Methods of Body Temperature Measurement between Different Anatomical Locations with Sedentary Dogs

The relationship between the various BT methods and locations were analyzed by linear regression, and correlation data are listed in Table 1. Based on all the resting daytime BT measurements, BT_eye_ and BT_ear_ were significantly (*p* < 0.0001), albeit only moderately, related to BT_rec_ (*r* = 0.381 and *r* = 0.615, respectively). Comparison of BT_eye_ to BT_rec_, or BT_ear_ to BT_rec_, indicated that BT_ear_ was similar to BT_rec_ at 09:00 and 11:30 with less than 0.26% (0.1°C) difference in recorded temperatures but underestimated BT_rec_ by 0.8% (0.3°C) at both 12:30 and 14:30, respectively (Table 2). BT_eye_ underestimated BT_rec_ by 1.9% up to 3.3% across all measurement times (Table 2). On average, BT_eye_ was approximately 1.5 or 1.7°F cooler compared to BT_ear_ and BT_rec_, respectively, for all four daytime measurement occasions and breed data combined.

**Table 1:** **Correlations between measurement locations for data acquired while dogs were sedentary or with exercise**.

Dependent variable	*r*	*p*-Value
**Daytime and sedentary body temperature**		
Eye versus rectal	0.381	<0.0001
Ear versus rectal	0.615	<0.0001
Eye versus ear	0.458	<0.0001
**Body temperature before and after exercise**		
Eye versus rectal	0.674	<0.0001
Ear versus rectal	0.735	<0.0001
Eye versus ear	0.640	<0.0001

**Table 2:** **Mean (±SD) temperature measured at various body locations of dogs for comparison between methods within a measurement time at different times of day while sedentary or relative to exercise**.^a^

Measurement sites	Time of day			
Daytime measures at rest	09:00	11:30	12:30	14:30
Rectal	38.3 ± 0.5	38.1 ± 0.5	38.0 ± 0.4	38.0 ± 0.4
Ear	38.4 ± 0.6	38.1 ± 0.5	37.7 ± 0.5	37.7 ± 0.5
Eye	37.5 ± 0.8	37.3 ± 0.9	36.7 ± 0.8	36.9 ± 0.9

	**Pre-exercise**	**Post-exercise**		
**Daytime measures before and after exercise**	**−30 min**	**0 min**	**15 min**	**30 min**

Rectal	38.3 ± 0.5	39.7 ± 0.9	38.8 ± 0.7	38.3 ± 0.6
Ear	37.5 ± 0.8	39.2 ± 1.1	38.4 ± 0.6	37.9 ± 0.7
Eye	37.5 ± 1.1	39.9 ± 1.3	38.7 ± 0.9	38.4 ± 1.0

*^a^Data are representative of combining data from both Labrador Retriever and Beagles*.

**Figure 2:**
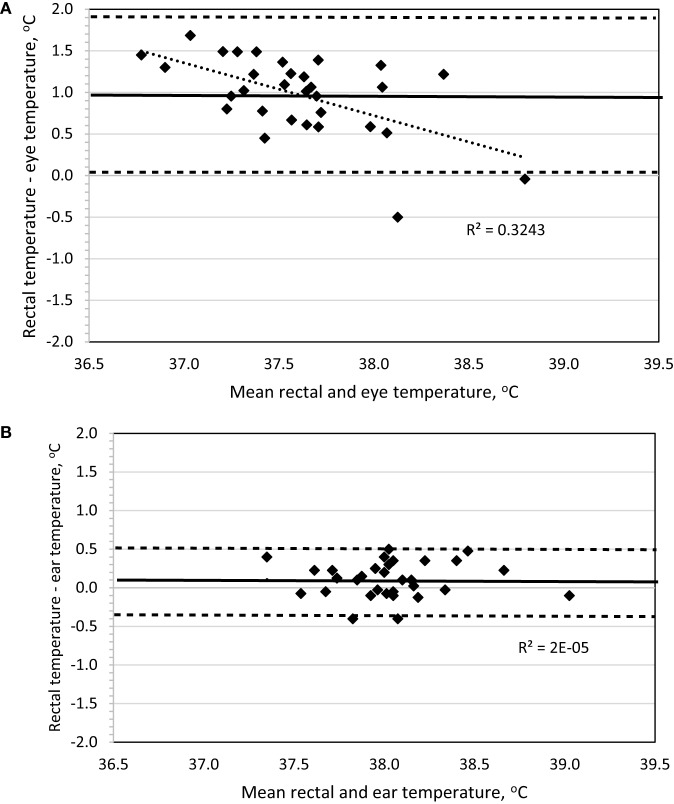
**Bland–Altman plots of body temperature in sedentary Labrador Retrievers or Beagles comparing (A) rectal and eye or (B) rectal and ear**. In both panels, the thin dotted line is the line of best fit for the data, the thick solid horizontal line is the mean difference (bias), and the horizontal dashed lines are the 95% limits of agreement (mean difference ± 1.96 SD). Points located above 0 on the *y*-axis represent an underestimation of temperature by the method in comparison to rectal core body temperature as measured by digital thermometer.

To determine method bias without measurement replication, the four daytime recordings were combined for each dog, and the mean was used for analysis. Evaluation of a Bland–Altman plot revealed that a significant bias (*p* < 0.001) existed for the IRT method measuring BT_eye_ to underestimate BT_rec_, the slope significantly (*p* < 0.001) differed from 0 with increasing underestimation at lower temperatures, and the 95% limit of agreement was ±0.925 (Figure 2A). By contrast, no bias was observed (*p* = 1.0) between the IRT method of BT_ear_ and BT_rec_, the slope was not different (*p* = 0.98) from 0, and the 95% limit of agreement was ±0.453 (Figure 2B).

### Exercise-Related Locomotor Activity and Body Temperature

Body temperature was measured before and at various times after a 30-min bout of play-exercise to characterize the effect of exercise on changes in BT between the breeds and at the same three anatomical sites as previously measured. Locomotor activity counts were recorded during the 30 min bout of play-exercise to assess exercise-related activity as a potential factor influencing BT. Activity counts did not differ (*p* = 0.53) between breeds (mean counts ±SE; Beagles = 42,700 ± 2,680; Labs = 40,500 ± 2,300). Ambient temperature of the indoor play area ranged from 25.6 to 26.7°C (78–80°F) for all groups of dogs.

**Figure 3:**
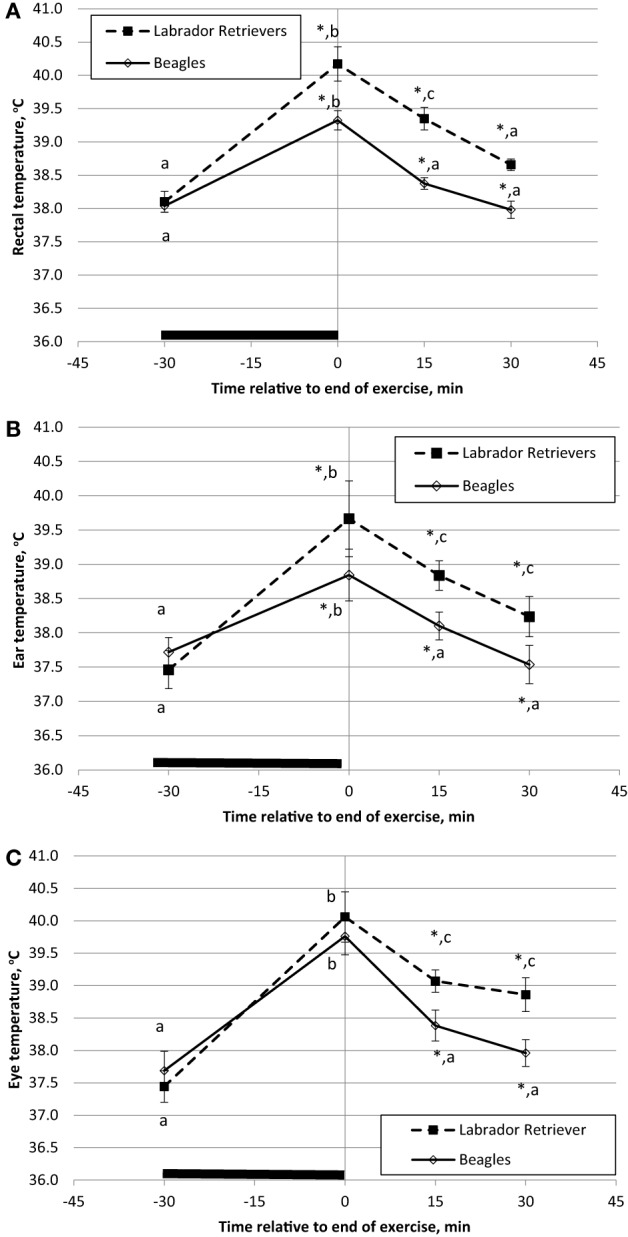
**Phase 1 mean (±SE) body temperature before and after play-exercise in Labrador Retrievers or Beagles**. **(A)** rectal body temperature, **(B)** ear temperature, and **(C)** eye temperature. Differences between breed (*p* < 0.05) at a given time have a *. Differences (*p* < 0.05) within a breed and between measurement times are indicated by letters (a, b, and c).

Temperature measures for each method were analyzed separately for ANOVA to evaluate the main effects of breed and time. BT in Celsius degrees is plotted separately for each breed over time with data from rectum (Figure 3A), ear (Figure 3B), or eye (Figure 3C). For both BT_rec_ and BT_ear_, a significant effect was observed for the interaction of time and breed in response to exercise (ANOVA *p* = 0.035 and *p* = 0.005, respectively). LS means for pre-exercise BT_rec_ and BT_ear_ were not different (*p* = 0.38 and *p* = 0.27, respectively) between breeds. On average, immediate post-exercise BT_rec_ and BT_ear_ significantly (*p* < 0.001) increased from pre-exercise temperature (Figures 3A,B) by 1.3 and 1.1°C, respectively, in Beagles or 2.0 and 2.3°C, respectively, in Labs. In addition, both BT_rec_ and BT_ear_ differed between breed (*p* = 0.001 and *p* = 0.001, respectively) with Labs measured to have higher BT_rec_ and BT_ear_.

A significant (*p* < 0.01) post-exercise recovery of BT_rec_ and BT_ear_ was observed in both breeds by 15-min. Furthermore, BT_rec_ and BT_ear_ recordings in Beagles at 15-min post-exercise had recovered to initial pre-exercise temperatures (*p* = 0.34 and *p* = 0.43, respectively). By contrast in Labs, although BT_rec_ and BT_ear_ had significantly decreased from post-exercise peak, both BT_rec_ and BT_ear_ recordings were still significantly higher (*p* < 0.01) from initial pre-exercise recordings by at least 1.1°C. By 30 min of recovery from exercise BT_rec_ had recovered to pre-exercise values, but BT_ear_ was still significantly elevated 0.9°C.

A marginally significant effect for BT_eye_ was observed for the interaction of time and breed in response to exercise (ANOVA *p* = 0.09). LS means for pre-exercise BT_eye_ were not different (*p* = 0.48) between breeds with minimal numerical difference by approximately by 0.3°C. For general comparison purposes, it is important to note that pre-exercise BT recordings were obtained at different times of day for each exercise group between 08:00 and 14:00. By contrast, during the sedentary study, the first BT recording was obtained for all dogs at approximately the same time of day (09:00). Immediate post-exercise, BT_eye_ significantly (*p* < 0.001) increased by 2.1 or 2.8°C in Beagles or Labs, respectively, from pre-exercise temperature. Although the main effect of breed for BT_eye_ was not significant (*p* = 0.18) and both breeds had similar locomotor activity counts over the 30-min exercise bout, on average Labs had a BT_eye_ of 40.3°C, which was approximately 0.5°C higher compared to Beagles. Linear regression analysis of the total exercise activity counts and the immediate post-exercise BT_eye_ revealed a significant, but only moderate, positive relationship (*r* = 0.35; *p* = 0.05).

Similar to BT_rec_ and BT_ear_, by 15-min post-exercise BT_eye_ significantly (*p* < 0.004) recovered in both breeds, as Beagles significantly declined by 1.4°C and Labs that declined by 1.2°C. Again, average Beagle BT_eye_ at 15 and 30-min post-exercise were similar to the pre-exercise readings, whereas Labs were still significantly elevated (*p* < 0.01) by 1.3°C after 30-min of recovery.

### Comparison of Methods of Body Temperature Measurement between Different Anatomical Locations with Exercising Dogs

The relationship between the various BT methods and locations were analyzed by linear regression, and correlation data are listed in Table 1. The relationship of BT measures between methods in response to play-exercise improved considerably compared to measurements recorded on rest days. Based on all the exercise BT measurements, BT_eye_ and BT_ear_ were significantly (*p* < 0.0001) related to BT_rec_ (*r* = 0.674 and *r* = 0.735, respectively). On average, BT_eye_ at all post-exercise times differed by less than 0.2°C compared to rectal temperatures, but BT_eye_ at pre-exercise underestimated BT_rec_ by 1.4% (0.5°C; Table 2). By contrast, BT_ear_ underestimated BT_rec_ by 1.0% (0.4°C) to 1.3% (0.6°C) across all measurement times (Table 2).

**Figure 4:**
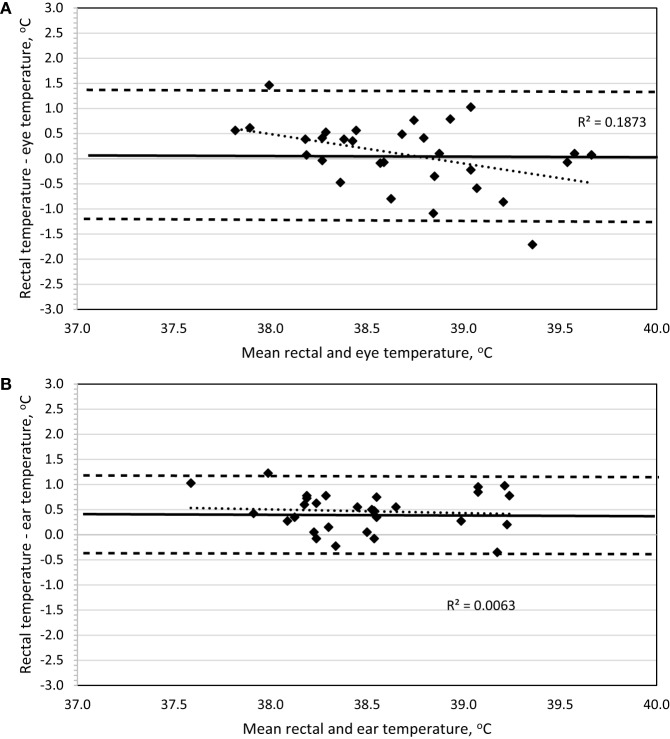
**Bland–Altman plots of body temperature in Labrador Retrievers or Beagles before and after 30-min of play-exercise comparing (A) rectal and eye or (B) rectal and ear**. In both panels, the thin dotted line is the line of best fit for the data, the thick solid horizontal line is the mean difference (bias), and the horizontal dashed lines are the 95% limits of agreement (mean difference ± 1.96 SD). Points located above 0 on the *y*-axis represent an underestimation of temperature by the method in comparison to rectal core body temperature as measured by digital thermometer.

To determine method bias without measurement replication, the four recordings were combined for each dog, and the mean was used for analysis. Evaluation of a Bland–Altman plot revealed that a significant bias (*p* = 0.02) exists between the IRT method of BT_eye_ and BT_rec_, and the slope is significantly different from 0 (*p* = 0.02) such that BT_eye_ will underestimate BT_rec_ at lower temperatures and overestimate BT_rec_ with increasing temperatures (Figure 4A). No bias (*p* = 0.67) exists between the IRT method of BT_ear_ and BT_rec_, the 95% limit of agreement was ±0.76, and the slope is not different (*p* = 0.62) from 0 (Figure 4B).

## Discussion

The primary focus of this study was to evaluate hyperthermia in response to play-bout exercise with the use of IRT methods to measure eye and ear temperature of dogs. We report that both eye and ear temperature significantly relate to rectal BT and uniquely demonstrate that both are sensitive to accurately detect hyperthermia changes associated with exercise. In addition, this research also demonstrates that ear measures in the resting dog are sensitive and accurate, whereas eye temperature, while sensitive, is considerably less accurate when related to rectal temperature.

Prior to pursuing the primary objective, 16 Labrador Retrievers and 16 Beagles were initially used to characterize multiple daytime eye and ear temperature during sedentary activity relative to rectal temperature as the best estimate of core BT (20). To achieve the primary objective, the same dogs were exercised for 30 min and temperature of the eye, ear, and rectum measured before and after exercise. Accurately monitoring core BT during exercise in the field is critical and one of the ways to assess the risk of heat stress (4, 21) and exercise intolerance (22, 23). However, to obtain a rectal reading, exercise must be halted, measurement requires temporary restraint, and measurement recording may be challenging because of refusal by the pet. Thus, monitoring BT at alternative body sites or using different detection methods could address some of the challenges with rectal temperature measures.

Past research has reported that tympanic membrane and hypothalamus share blood supply from the carotid arteries (24, 25), and a reading is reflective of heat from both tympanic membrane and ear canal (26). Areas of the eye, especially around the posterior border of the eyelid and the lacrimal caruncle, have rich capillary beds that respond to changes in blood flow resulting in localized temperature changes in people (27), cattle (28), and dogs (8, 29). Therefore, eye and ear temperature in sedentary and exercising Beagles and Labrador Retrievers are not only the surrogates of brain temperature but also reasonable alternatives to assess core BT fluctuations.

This study evaluating sedentary dogs indicated that ear temperature is better related (*r* = 0.615) than eye temperature (*r* = 0.381) when compared to rectal temperature as the reference method. When examined to monitor hyperthermia after exercise, the relationship for both ear (*r* = 0.735) and eye (*r* = 0.674) temperature compared to rectal temperature improved considerably (Table 1). The results generated from sedentary dogs confirm previous observations that ear and eye temperature significantly relate to and underestimate rectal BT (10, 11, 20).

Based on all daytime measurements, ear temperature underestimated rectal temperature on average by 0.1–0.3°C, which is similar to previous reports using infrared auricular thermometers versus rectal temperatures in dogs (10, 11). This underestimation increased to 0.4–0.6°C when measured to assess hyperthermia following exercise. Regardless of this minor loss of accuracy, Bland–Altman plot analysis indicates there is significant agreement, thus no bias, between the measurements of ear and rectal temperatures when measuring sedentary or hyperthermic BTs. In addition, sedentary data revealed that the 95% limit of agreement of ±0.45 is below the suggested, albeit arbitrary, limit of ±0.5 utilized in veterinary-based method evaluation (6, 10, 11).

A secondary goal was to explore the use of a remote-detection method of measuring eye temperature based on IRT to eliminate the need to insert a thermometer probe into the ear or rectum. This would potentially serve the dual benefit of improved pet compliance and ultimately reduce the need to halt the dog while working or exercising. As stated above, the relationship between eye (*r* = 0.674) temperature compared to rectal temperature improved considerably when measured for exercise-induced hyperthermia. Other canine studies evaluating eye temperature with IRT indicate a similar relationship between eye and rectal measures [*r* = 0.661; Ref. (8)]. However, evaluation of eye temperature in the sedentary dog revealed a greater underestimation of rectal temperature (Table 2) versus ear temperature and resulted in a broader 95% limit of agreement (±0.925), which worsened when used to measure BT after exercise. This indicates that eye temperature should not be used interchangeably with rectal temperature but still appears to be a feasible methodology and anatomical location to effectively monitor exercise-induced hyperthermia.

The daytime temperatures measured between 09:00 and 14:30 were obtained while the dogs were considered sedentary and did not exhibit the natural diurnal fluctuation of BT that normally increases 0.5–0.8°C from early morning (08:00–10:00) and peaks between 17:00 and 20:00 (17, 30, 31). This lack of diurnal fluctuation was observed across all three methods, and as shown in Figure 1, the temperatures were relatively consistent or slightly declined over the day. This observation is likely reflective of a natural increase in daytime BT because the dogs were in a kennel facility, slightly excited by entrance of familiar pet-care staff, and temporarily walked out of the kennel area for BT measures. Since the goal of the study was to examine BT methodology in sedentary and then in response to exercise, precautions such as temporary housing in metabolism cages to minimize external stimulation were excluded from the study design, thus preventing observation of the subtle diurnal fluctuation.

Finally, the third goal was to evaluate BT fluctuations between dogs of different body size to determine if breed differences were apparent between the three different temperature measurement methods. This study also reports observations of breed size differences in BT with all three methods resulting in Labrador Retrievers having a higher BT compared to Beagles, with no observed interaction between breed and time in the sedentary portion of the study. Other studies have reported an opposite observation of small breed dogs having a higher BT compared to larger breed dogs (17). It is unclear of the discrepancy between breed sizes between this study and others. However, other research by our lab has observed that Labrador Retrievers exhibit higher BT compared to similarly sized dogs (Shepherd breeds; Zanghi and Otto, unpublished data). Therefore, it appears to be important to consider the actual breed, and not just relative breed size, when considering “normal” BT ranges. Furthermore, breeds like Labrador Retrievers may be more prone to elevated BT, thus more at risk of heat stress, during exercise at higher ambient environmental temperatures.

Piccione et al. (17) did observe breed differences with diurnal change in daytime and nighttime BT, which, as stated above, is sensitive to masking because of physical activity or environmental stimuli. Thus in the current study, the movement of the dogs in the AM to obtain the measurements may have influenced the morning BTs that was sustained throughout the day with subsequent measurement collection. If so, Labs in this study appear to be more sensitive to external stimulation, and/or exercise that causes a rise in BT. The breed difference was consistent even when BT was measured in response to exercise. Therefore, it is difficult to attribute physical movement of the dogs during the sedentary study as the primary confounding factor, as exercise activity counts were similar between Labs and Beagles, but all three BT methods generated data that resulted in a significant or marginally significant breed × time interaction with Labs having a higher BT compared to Beagles. It is possible that the Labs respond differently with exercise that may be a result of lower thermal dissipation, which results in a higher BT.

In conclusion, both eye and ear temperature are effective for monitoring exercise hyperthermia, which is significantly related to rectal BT. However, eye temperature should not be used interchangeably with rectal temperature. This initial study provides evidence supporting the use of IRT measurement of eye temperature as an effective method of monitoring BT in a passive manner and remotely without having to physically contact the dog. Therefore, using a remote and passive eye temperature technology appears to address some of the challenges of pet restraint and pet compliance during probe insertion. It should be noted that although these data are representative of hyperthermia during exercise, exercise was only moderate rigorous, and average BT measures only reached approximately 40°C (104°F). Thus, additional research is warranted to examine the use of eye temperature in dogs experiencing higher BTs, in which remote detection of eye temperature of a dog approaching a higher risk of heat stress would be of significant value to the health of the pet. Finally, because this IRT device was deemed industrial-grade and used for research purposes, additional research to examine other similar devices, but possibly less sensitive and less expensive, would be valuable and likely more feasible for widespread use.

## Ethics Statement

Study protocol was designed and followed in strict accordance with the guidelines established by the Nestlé Purina PetCare Animal Care and Use Advisory Committee at the pet-care facilities of Nestle Purina PetCare.

## Author Contributions

The author was the primary person responsible for designing the study, analyzing the data, and drafting the manuscript.

## Conflict of Interest Statement

The author would like to indicate that funding was solely provided by Nestle Purina Research and that the author is employed by Nestle Purina PetCare. No other conflict of interests exists, and all temperature and activity devices were purchased from the manufacturers. The data included in the manuscript have not been previously published, and animal protocols were conducted in accordance with approved Animal Care and Use Committee protocols.
